# Image-guided minimally invasive treatment for small renal cell carcinoma

**DOI:** 10.1007/s13244-018-0607-4

**Published:** 2018-04-06

**Authors:** Miltiadis E. Krokidis, Panagiotis Kitrou, Stavros Spiliopoulos, Dimitrios Karnabatidis, Konstantinos Katsanos

**Affiliations:** 1 The Department of Radiology, Cambridge University Hospitals NHS Foundation Trust, Hills Road, Cambridge, CB2 0QQ UK; 2 The Department of Interventional Radiology, Patras University Hospital, School of Medicine, 26504 Rion, Greece; 3 The 2nd Department of Radiology, Interventional Radiology Unit, ATTIKO Athens University Hospital, 1st Rimini St, Chaidari, GR 12461 Athens, Greece; 4 The Department of Interventional Radiology, Guy’s and St. Thomas’ Hospitals, NHS Foundation Trust, King’s Health Partners, London, SE1 7EH UK

**Keywords:** Small renal tumours, Renal cell carcinoma, Radiofrequency ablation, Microwave ablation, Cryoablation

## Abstract

**Abstract:**

Surgical partial nephrectomy is still considered as the “gold standard” for the definitive management of small malignant renal masses, whereas treatment with image-guided percutaneous ablation is still mainly reserved for those patients who cannot undergo nephron-sparing surgical resection due to advanced age, underlying comorbidities or compromised renal function. Nonetheless, the recent evidence that underlines the long-term oncological equipoise of percutaneous ablation methods with surgical resection in combination with the reduced complication rate and cost supports the use of an image-guided minimally invasive approach as a first-line treatment. The purpose of this review is to offer an overview of the most widely used percutaneous renal ablation treatments (radiofrequency, microwave and cryoablation) with a focus on their main technical aspects and application techniques for curative ablation of small renal cell carcinoma (stage cT1a). The authors also provide a critical narrative of the relevant medical literature with an emphasis on outcomes of comparative effectiveness research, and appraise the percutaneous methods compared to surgery in the context of evidence-based practice and future research studies.

**Teaching Points:**

• *RCC is a common cancer and is increasingly detected incidentally at early stages.*

• *There is long-term oncological equipoise of percutaneous ablation compared to surgical resection.*

• *Large-scale trials are required to produce Level 1a evidence.*

## Introduction

Renal cell carcinoma (RCC) is among the ten commonest tumours among both males and females in the western world [1]. The increased incidental detection rate of small asymptomatic renal tumours is a result of the higher number of cross-section scans that are performed for other reasons [2]. More than 80% of those small renal masses (SRMs) have proved to be cT1a (maximum diameter smaller than 4 cm) renal cell carcinomas (RCCs) [3]. The management of such small lesions generates significant controversy; the potential options are active surveillance and nephron-sparing treatments with either surgery or ablation [4, 5]. Indications for treatment may be absolute (i.e. patients with only one anatomical or functional kidney) or relative (i.e. patients with hereditary forms of renal cell carcinoma, functioning opposite kidney that is affected by a disorder that might impair renal function in the future) [6, 7]. Surgery has evolved from open radical to partial nephrectomy (PN) and the latter may be performed as open, conventional laparoscopic or robot-assisted laparoscopic. Nephron-sparing surgery in tertiary care centres may offer a 5-year survival of up to 90% with satisfactory preservation of renal function and reduction of the frequency of cardiovascular events in comparison to the radical excision [8–11]. Active surveillance (AS), on the other hand, has been historically reserved for those patients who may be unfit for surgery. The concept of AS is based on the fact that the growth rate of the majority of these tumours is relatively slow (2-3 mm/year) and the metastatic potential is also, in general, rather low. However, given that there are currently no biomarkers that may predict the natural history of such masses, close monitoring with cross-sectional imaging is required in order to detect those tumours that will grow rapidly [12–14]. Furthermore, the Swedish Kidney Cancer Quality Register has highlighted that there is a more significant metastatic potential than what is generally believed, even for tumours that are between 1 and 4 cm [15].

The image-guided percutaneous ablation techniques and mainly radiofrequency ablation (RFA) and cryoablation have been applied in the management of SRMs in the last 15 years and offered very promising clinical results.

The present review offers an overview of the percutaneous ablation treatments available to date, focusing on their main technical aspects and application techniques for curative ablation of small RCC. The authors also provide a critical narrative of the relevant medical literature with an emphasis on long-term outcomes of comparative effectiveness, and appraise the percutaneous methods compared to surgery in the context of evidence-based practice and future research studies. Clinical data are presented both for long-term oncological outcomes and for the preservation of renal function compared to the historical standard of partial nephrectomy.

## Percutaneous approach

Percutaneous ablation is a minimally invasive, image-guided procedure, performed most of the time under conscious sedation. Guidance may be under ultrasound (US), computed tomography (CT) or magnetic resonance imaging (MRI) and aims at the accurate and precise placement of the energy electrode into the target tumour. Patient preparation typically involves optimisation of clotting factors, if necessary, and overnight fasting to allow for safe administration of intravenous conscious sedation. An absolute pre-requisite is sufficient coagulation values (INR <1.45) and platelet count (>50,000/μl). After informed consent, access planning and local anaesthesia of the skin and subcutaneous tissues is performed under aseptic conditions. The lesion should have been previously biopsied in another session.

Some procedures may be performed as day cases, but routinely the patient will stay overnight in the hospital for routine observations and a follow-up scan the next day to confirm the immediate result and exclude any treatment-related complication; i.e. haematoma or bowel injury. In line with the principle of R0 surgical excision, an R0 ablation with enough tissue margin to minimise the risk of residual tumour or early recurrence is advocated at all times [16]. Still, the ability to easily repeat the procedure if necessary, coupled with the fact that one of the most important features of percutaneous renal ablation is its nephron-sparing nature and renal function preservation; in general, a conservative but adequate ablation margin will suffice, especially in higher risk cases with solitary kidneys or already impaired renal function at early stages of chronic kidney disease [17]. Interventional radiologists performing percutaneous renal ablation procedures have both the advantage of regular imaging follow-up post-procedure, and the ability of repeating the procedure should this be necessary at some point in the future.

## Percutaneous ablation methods

### Radiofrequency ablation (RFA)

RFA was first reported in liver tumours in the 1990s [18], and is the most widely used and most investigated method for the treatment of small renal masses [17, 19]. RFA uses an alternating current (460–500 kHz) delivered through the lesion with the use of straight or expandable applicators known as electrodes. RFA requires the use of grounding pads on the skin to allow for a closed electrical circuit between the body and the RFA generator. Circulation of the alternating current causes high-frequency agitation of the ionic molecules contained within the tissues and thereby frictional heat is produced. Temperatures between 60 °C and 100 °C produce immediate cell death by protein denaturation and coagulative tumour necrosis. However, in temperatures above 100 °C, water vaporises and tissue adjacent to the electrode may carbonise, this way degrading the electrical conductance properties and increasing tissue impedance; the latter may result in a suboptimal treatment effect; electrodes with a circulating cooling saline chamber have been developed to limit this phenomenon. The efficacy of RFA is also limited by adjacent high-flow vascular structures, producing the well-known energy-sink or heat-sink effect; this occurs more frequently in the liver where major vascular structures may be located adjacent to the ablated areas, whereas in the kidney it may be encountered only for the very central tumours.

### Microwave ablation (MWA)

There has been a recently increasing interest about MWA for SRMs [20]. MWA was developed to overcome the major limitations of RFA in the liver; i.e. the heat-sink effect. Microwave technology is based on the application of an electromagnetic wave (915–2,450 MHz) through an antenna that causes the tissue water molecules to rotate and re-orientate, thus increasing their kinetic energy resonating with the applied waves. The latter produces heat and increases tissue temperature to nearly 100 °C. Compared to RFA, no grounding pads are necessary and a larger area is ablated as higher temperatures are reached within the tumour regardless of tissue electrical conductance. Moreover, MWA operates independently of any electrical current convection and is not limited by tissue impedance, desiccation or charring and heat sink phenomena. The most recent microwave generators using the higher frequency of 2,450 MHz are producing more homogeneous and more spherical ablation results without the “rat-tail” ovoid shape ablation results of the first generation 9.15-MHz devices [21, 22]. In addition, internally cooled antenna shafts have been developed to enhance efficacy at higher energies without collateral damage. Hence, 2,450-MHz devices with internal cooling are becoming the norm nowadays for the predictable ablation of tumours up to 4–5 cm in a variety of solid organs.

### Cryoablation

Cryoablation is an ablative technique that causes tumour necrosis by freezing. For most types of tissues, cellular necrosis occurs when the temperature reaches -20 °C, due to ice formation and osmosis causing protein denaturation, cell membrane rupture and cell death. Temperature below -10 °C is delivered to the tissues within seconds due to rapid, high-pressure (300 bars) argon gas expansion by the Joule-Thomson effect [16]. This is achieved with the use of percutaneously inserted argon-gas cryoprobes causing controlled tissue freezing; the effect may be monitored under CT or MRI as a so-called “ice-ball”. Cryoablation sessions usually involve two cycles of 10 min freezing and 10 min thawing (achieved by the circulation of helium gas). During the last freezing phase, intracellular ice is formed leading to cellular membrane rupture and cell death [23]. In addition, there is an indirect ischaemic insult, because of microvascular occlusion occurring during the thawing phase of the cycle [24].

Cryoablation is limited by the cool-sink effect when high-flow vascular structures are in the vicinity of the tumour and disrupt the aforementioned energy exchange of the “ice-ball”, similarly to the heat-sink effect for RFA. Current cryoablation devices offer 17-G cryoprobe needles, each of which may produce an ablative “ice-ball” of around 3 cm in the short axis; hence, multiple cryprobes need to be inserted and activated concurrently for the treatment of larger tumour lesions [25]. Cryoablation is particularly elegant for the treatment of centrally located renal tumours that may abut or even infiltrate the collecting system because of its minimal risk of thermal injury. Cryoablation is also favoured regarding the curative destruction of renal tumours larger than 4 cm or those that are very close to the spinal column or the bowel, because of its ability to produce complex volumetric ablation results with multiple overlapping cryoprobes; furthermore, the ablation result may by monitored in real time taking into account the isothermal properties of the “ice-ball” [26, 27].

## Outcome measures

There are certain important and fundamental outcomes that percutaneous image-guided ablation methods need to achieve, in order to accomplish equipoise with the surgical nephron sparing. The main clinical outcome measure is oncological, i.e. disease-free or cancer-free survival—defined as freedom from local recurrence and/or remote metastasis and cancer-related patient death, respectively [2]. Furthermore, renal function preservation and peri-procedural complication rates of surgical and percutaneous methods need to be assessed in order to complete decision-making and patient management [17]. Amassed clinical evidence to date provides data about the performance of percutaneous renal ablation methods mainly at the bottom level of the evidence pyramid; that is mostly single-arm observational cohort studies or comparative unmatched studies with population-based comparator groups, and some large case series that are available so far, together with some relevant meta-analyses based on these studies and with only one randomised controlled trial (RCT) in the case of MWA [3, 17, 20, 24, 28]. However, as evidence is mounting there is increasing confidence in the robustness of the clinical results, the minimally invasive nature, the better preservation of renal function and the increased cost-effectiveness of percutaneous methods compared to surgery. The authors will analyse each individual outcome measure separately.

## Peri-procedural complication rates

A proportional rate meta-analysis of single-centre reported outcomes in nearly 10,000 patients has previously shown that in case of cT1a tumours RFA is associated with significantly lower rates of major complications (3.1%) versus open (7.9%) or laparoscopic/robotic partial nephrectomy (7.2%, both < 0.001). On the contrary, minor complications were encountered significantly more frequently in case of RFA (13.8% vs 7.5–9.5%, *p* < 0.001) [29]. In a comparative effectiveness meta-analysis of six clinical studies with 587 patients in total, comparing thermal ablation (RFA and MWA) to partial nephrectomy, Katsanos et al. [3] reported that overall complication rates were significantly lower in the ablation group (7.4% vs 11.0%; RR, 0.55; 95% confidence interval [CI], 0.31–0.97; *p* = 0.04) and the same trend was noted in case of major complications as well (RR, 0.45).

Yin et al. [30] recently published an updated meta-analysis that synthesised 12 comparative studies evaluating RFA against partial nephrectomy and including 2,358 patients in total. The pooled analysis showed no significant difference between RFA and PN with respect to major complication rate (3.7% vs 4.4%; RR, 0.83; 95% CI, 0.43–1.60; *p* = 0.579). However, a slightly more expanded meta-analysis by Pan et al. [31] with 16 studies in total focusing again on percutaneous RFA (excluding MWA and cryoablation) compared with PN reported numerically lower major and minor complication rates in favour of RFA (major: odds ratio [OR], 0.74; minor: OR, 0.45, respectively). With regards to the method of ablation, a pooled proportion meta-analysis of 31 case-series studies (20 cryoablation with 457 cases and 11 RFA with 426 cases) reported no significant difference in the rate of complications between RFA and cryoablation options [32]. Aggregate data on complications relating to renal MWA is sparse, but anecdotally MWA is considered to be as safe as RFA in case of SRM treatments.

## Renal function impairment

Impaired baseline renal function is a common condition in patients with SRMs, especially in the case of advanced age or other underlying comorbidities like diabetes. Preservation of renal function is, therefore, of paramount importance for patients undergoing any kind of nephron-sparing treatment, especially in cases where renal function is already impaired. For such patients, sparing haemodialysis is crucial for the preservation of quality of life [33]. The main advantage of the percutaneous methods on that aspect is the lack of necessity of clamping the main renal artery, therefore avoiding the warm-ischaemia time that is required with all types of surgical excision of renal masses [34, 35].

Katsanos et al. [3] reported a significantly lower GFR decline in case of percutaneous thermal ablation (mean difference, −14.6 ml/min/1.73 m^2^; 95% CI, -27.96 to −1.23; *p* = 0.03). Results were in line with the meta-analysis by Yin et al. [30] (lower eGFR decline after RFA—weighted mean difference [WMD], -3.90; 95% CI, -6.660 to −1.140; *p* = 0.006), and further corroborated by Pan et al. [31]. The latter reported that baseline eGFR was lower in the RFA arm (because of apparent patient selection; WMD, -7.27; 95% CI, -11.99 to −2.55; *p* = 0.003) and post-operative decline was also significantly lower in case of RFA compared to PN (WMD, -4.82; 95% CI, -9.33 to −0.31; *p* = 0.04). Interestingly, a recently published comparison between 63 percutaneous RFA and 63 robotic PN cases reported very similar findings with significantly higher deterioration of renal function in case of surgery. Post-procedure renal function decline at 30 days was significantly less in RFA [(−0.8) ± 9.6 vs (−16.1) ± 19.5 ml/min/1.73 m^2^; *p* < 0.0001) [17].

## Freedom from local recurrence and metastasis

A large retrospective analysis was recently published by Thompson et al. [36] comparing PN to thermal ablation methods for the treatment of stage cT1 RCC (stages cT1a and cT1b). This single-centre study included 1,803 patients (1,424 cT1a and 379 cT1b) from the Mayo Clinic over a period of 12 years. In the majority of cases, patients were treated with PN (1,383 patients), while 180 underwent RFA and 240 cryoablation. In this analysis there was no significant difference in local recurrence-free survival rate between the three treatments (*p* = 0.49). Metastasis-free survival was significantly better for PN (*p* = 0.005) and cryoablation (*p* = 0.021) compared to RF for cT1a tumours. No patients with cT1b tumours were treated with RFA. As this analysis extends back to more than a decade, there is a clear selection bias in patients chosen for treatment with ablating methods, which were significantly older and with inferior life expectancy (*p* < 0.001).

The comparative meta-analysis by Katsanos et al. [3] (synthesis of six studies) focusing on thermal ablation (RFA and MWA, excluding cryoablation) compared to PN reported no significant differences with regard to local recurrence rate (3.6% vs 3.6%; hazard ratio [HR], 0.92; 95% CI, 0.4–2.14; *p* = 0.79) or overall disease-free survival (HR, 1.04; 95% CI, 0.48–2.24; *p* = 0.92). Yin et al. [30] reported similar pooled results (synthesis of 12 studies) with no virtual difference in the frequency of local recurrence (4.14% vs 4.10%; RR, 1.18; 95% CI, 0.68–2.07; *p* = 0.550) and distant metastases (2.76% vs 1.89%; RR, 1.31; 95% CI, 0.70–2.46; *p* = 0.686).

Pan et al. [31] (synthesis of 16 studies evaluating RFA versus PN) reported slightly differing pooled results with more local recurrences in case of RFA (OR, 1.81; 95% CI, 1.14–2.88; *p* = 0.01), but no difference in the case of distant metastases (OR, 1.63; 95% CI, 0.74–3.58; *p* = 0.22). Arguably, Pan et al. did not include some of the studies available in the previous two meta-analyses, and most importantly, applied the odds ratio as the outcome metric, which is usually not indicated for cancer-related outcomes. Hazard ratios (HRs) that account for variations in follow-up periods between unmatched patient populations are typically indicated for time-to-event summary analysis of oncological outcomes like cancer recurrence and metastasis.

Freedom from local recurrence and/or remote metastasis was retrospectively examined in another study, in a cohort of 63 RFA cases versus 63 robot-assisted PN cases and documented that local recurrence was numerically (not significantly) higher in the RFA group (6/63 vs 1/63, *p* = 0.11), without, however, any significant difference in terms of disease-free survival (adjusted HR, 0.6; 95% CI, 0.1–3.7; *p* = 0.60) [17].

## Cost-effectiveness analysis

Due to minimal invasiveness, percutaneous ablation treatments may be performed as day-cases and in significantly less operating time. Pan et al. [31] reported that RFA was associated with shorter length of hospital stay (WMD, −2.02 days; 95% CI, -2.82 to −1.22; *p* < 0.001). Yin et al. [30] found an identical result with significantly shorter hospital stay in case of RFA as well (WMD, −2.02 days; 95% CI, -2.77 to −1.27; *p* < 0.001). In a cost comparison study for nephron-sparing treatments for cT1a masses including 173 patients who underwent open PN, robot-assisted PN, laparoscopic RF ablation or CT-guided RF ablation, Castle et al. [37] concluded that the 6-month cost of nephron-sparing surgery is lowest with RFA by either a laparoscopic or CT-guided approach compared to open or robot-assisted PN. Another study on 46 patients by Lotan et al. [38] dated back in 2005, compared RFA with PN performed with open or laparoscopic surgery. The authors concluded that for SRMs of appropriate size, minimally invasive methods (RFA) can decrease morbidity, along with significant cost benefits. Percutaneous RFA was significantly cheaper to perform (US$4,454 ± 938), compared to both laparoscopic PN (US$7,013 ± 934) and open PN (US$7,767 ± 1,605) [38].

## Five-year projection

Building on the on-going engineering developments in the technology of percutaneous microwave and cryoablation systems, image-guided ablation of small renal cell carcinoma is destined to develop to the new standard of care for the nephron-sparing treatment of patients with localised stage cT1a tumours (<4 cm and without nodal involvement), owing to its minimally invasive, safer, cheaper and equally effective properties. Quality of life studies and patient-reported outcomes (PROs) are anticipated to confirm a more positive patient feedback in comparison with surgery, raise the profile and increase acceptance of percutaneous ablation methods by patient groups, and government and privately funded healthcare systems. Large-scale population-based or other comparative effectiveness studies comparing percutaneous ablation with open or laparoscopic or robotic partial nephrectomy are eagerly awaited to confirm the long-term non-inferiority of the technique, along with its favourable cost-effectiveness profile. In the field of image guidance, the authors expect gradual introduction of radiation-free MR-based or other optically guided navigation systems that will further improve the precision of electrode/antenna placement and the accuracy of the predicted ablation volumes, so as to virtually eliminate the rate of inadvertent complications, incomplete treatment and/or local tumour recurrence.

## Conclusions

In conclusion, renal cell carcinoma is a common cancer in both men and women, and is being increasingly detected at earlier stages as an incidental small renal mass. Surgical partial nephrectomy has been the historical “gold standard” nephron-sparing treatment, with percutaneous ablation methods reserved for non-surgical candidates because of advanced age, underlying comorbidities or already compromised renal function. Nonetheless, recent evidence underlines the long-term oncological non-inferiority of percutaneous ablation methods compared to surgical resection, coupled with its unique properties of a completely incisionless procedure, lower rate of complications, better preservation of renal function and more favourable cost-effectiveness profile compared to partial nephrectomy. Nevertheless, significant challenges and unmet clinical needs remain, as not all tumours are amenable to ablation or some may not be visible enough to be targeted successfully, and the level and quality of relevant clinical evidence remains low.
